# Beyond “implementation”: digital health innovation and service design

**DOI:** 10.1038/s41746-018-0059-8

**Published:** 2018-09-20

**Authors:** James Shaw, Payal Agarwal, Laura Desveaux, Daniel Cornejo Palma, Vess Stamenova, Trevor Jamieson, Rebecca Yang, R. Sacha Bhatia, Onil Bhattacharyya

**Affiliations:** 1 Institute for Health System Solutions and Virtual Care, Women’s College Hospital, 76 Grenville Street, Toronto, ON Canada; 2 Institute of Health Policy, Management and Evaluation, University of Toronto, 76 Grenville Street, Toronto, ON Canada; 3 Department of Medicine, University of Toronto, Toronto, ON Canada; 4 Division of General Internal Medicine, St. Michael’s Hospital, Toronto, ON Canada

**Keywords:** Health services, Translational research

## Abstract

Digital tools have shown great potential to enhance health services’ capacity to achieve the goals of the triple aim (enhance patient experience, improve health outcomes, and control or reduce costs), but their actual impact remains variable. In this commentary, we suggest that shifting from a perspective focused on “implementing” new digital tools in health care settings toward one focused on “service design” will help teams execute more successful digital technology adoption projects. We present value proposition design (VPD) as a service design strategy requiring that stakeholders are brutally honest in determining the value of a new digital tool for their everyday work. Incorporating a perspective focused on how the value proposition of a technology is understood by each team member, and implications for their work routines, will help project teams to better understand how services can be reinvented during technology adoption initiatives. We present the simple heuristic [Tool+Team+Routine] as a reminder of the central considerations that make up a service design initiative, and present an illustrative case scenario of designing the use of a digital care coordination platform in an actual digital technology adoption project. We conclude by outlining two important challenges that need to be addressed to advance service design approaches to technology adoption in health care.

## Introduction

Many digital health innovations show potential to improve health outcomes, reduce health system costs, and improve patient experience, but their impact remains variable and limited in scope.^1–3^ Commentators have addressed a variety of themes related to broader strategies for generating useful health innovations,^4,5^ and implementation science has illustrated a number of key considerations for introducing technologies into new settings of health care delivery.^6^ However, despite recent advances in implementation science for digital health, a central component of successful digital health innovation remains largely unaddressed in academic literature: the intimate connection between new digital tools and the changes they necessitate to the actual delivery of health care services. In other words, the inter-relation between *product innovation* and *service innovation* for digital health has not been sufficiently acknowledged, contributing to the ongoing challenges of technology adoption in health service delivery settings. In this paper we highlight the relationship between digital health tools, implementation science, and the practice of *service design*, outlining the ways in which an approach informed by service design can promote the more successful and sustainable deployment of digital tools in health care.

The central purpose of this paper is to clarify the changes to services that are required when new digital health tools are introduced into a health service delivery process. We outline the utility of attending to the “value proposition design” of a particular digital tool in a specific context of use,^7^ and introduce the simple heuristic [Tool+Team+Routine] as a method for understanding the implications of a technology for the actual delivery of health care services. We present a brief synopsis of this approach to offer concrete guidance for health care providers, managers, and policymakers in understanding the implications of new digital tools for the innovation in services that accompany them. In so doing, we explain how teams hoping to promote the use of digital health tools can use methods of service design that go beyond conventional approaches to implementing technology in particular settings of health care.

## Service design and the implementation process

To introduce the unique characteristics of an approach to implementing digital technologies in health care that is informed by service design, we begin with a discussion of objectives. The objective of service design is to carefully plan and promote the coordinated action required to execute a high quality health care service.^8,9^ Service design is not about the technology per se, but about the overall quality of the proposed configuration of service delivery that might result from the comprehensive adoption of a new technology.^10,11^ It is about reinventing the service process to achieve a greater (and often different *kind of*) impact, as opposed to simply improving existing processes and workflows. Furthermore, a service design perspective acknowledges that technologies are not fixed and immutable.^12^ Instead, they are always subject to revision and refinement based on emerging insights about their usability and effectiveness, and the evolving needs of particular settings.^13^

As described here, a service design approach explicitly acknowledges that the ideas and objects first introduced to a setting will need to evolve to meet the needs of the people adopting them. This point resonates strongly with the idea from implementation science that best practices have a “central core” and an “adaptable periphery”,^14,15^ and that interventions *should be* adapted to fit within particular health service environments. It also raises an important question about the extent to which best practices should be adapted to meet the needs of local adopters, and how far local adopters should be expected to change their own actions to adopt a new technology that represents an emerging best practice. We will address this important point later in this paper, but for now simply wish to emphasize the importance of considering the *service innovations* that accompany the adoption of any new technology.

Our emphasis on service design for digital health tools clarifies the importance of the *service innovations* that accompany product innovations. Where a new digital tool is successfully incorporated into a service delivery environment, we suggest that optimal impact generally arises when a new configuration of services occurs.^16,17^ For example, a digital care coordination platform may function to enhance the method by which patients and clinicians communicate, enabling video conferencing or text messaging to replace telephone calls and face-to-face visits in certain cases. However, the digital care coordination platform stands to make a much larger and different kind of impact when the broader approach to its deployment involves the active recruitment of a variety of health care providers to participate in ways that enable joint decision-making and more coordinated care delivery. The latter approach resonates with the principles of service design, incorporating an explicit focus on the role of the digital tool as one feature of a reinvented configuration of health services.^18^

## Service design explained

Service design is a concept that evolved fairly recently in the broader context of design practice, emerging especially through the work of design consultancy IDEO in the late 1990s.^9,19^ Service design applies “design principles”, the guiding concepts and frameworks originally developed from product design,^20^ to think through the meaning and experience of services for people who make up a given service system. This includes an explicit focus on end users, but it also includes a focus on those delivering the service and even those not physically present in a given service encounter.^9,20^ That means that the experiences and needs of managers, organizational leaders, health system funders, and other policy-level stakeholders are also all considered throughout the service design process.

Saco and Goncalves^9^ define service design through a series of four principles, suggesting that service design:

1. Aims to create services that are useful, useable, desirable, efficient, and effective
2. Is a human-centered approach that focuses on customer experience and the quality of service encounter as the key value for success
3. Is a holistic approach that considers in an integrated way strategic, system, process, and touch-point design decisions (i.e., decisions about the ways in which users actually interact with services)
4. Is a systematic and iterative process that integrates user-oriented, team-based interdisciplinary approaches and methods in ever-learning cycles

These principles of service design are highly customer-focused, but there is one important modification required to adapt them to health care environments. While customer experience is key to success in all services, in health care the service being delivered is also intended to achieve the potentially more important goal of improving, sustaining, and sometimes saving human life.

In health services, key outcomes are often framed as the Triple Aim of enhanced patient experience, improved population health outcomes, and controlled health care costs.^21^ In this way, service design in health care settings must balance the demand for excellent customer experience with the ability to achieve gains in the other domains of the Triple Aim, including the efforts to improve health and reduce cost. The ways in which a service design approach can leverage new digital tools to help achieve the goals of the triple aim depend on the specific contexts in which digital tools are being adopted into health care services. We now turn to outlining two common contexts for technology adoption, “technology-push” versus “demand-pull”, and then expand on the use of value proposition design as a particular strategy of service design.

## Two scenarios of technology adoption

Technology adoption in health care commonly occurs under one of two scenarios: “technology-push” or “demand-pull”.^22,23^ Technology-push scenarios are where a technology provider has negotiated either a pilot project or larger adoption of their technology in a particular health care environment, often with a manager or other decision-maker who will not interact directly with the product. In these cases, the people who will use the product have not yet bought into the value of the product before the decision to procure it is made. Generally speaking, this scenario makes adoption more challenging.

The demand-pull scenario is where a team of people representing a service delivery environment identify a clear problem they are facing in their service. They scope out the nature of the problem, and identify a particular kind of technology that could help to solve their problem. After this work has been done, the team then identifies a particular tool that meets an existing, well-defined need. This approach generally maximizes the ease of achieving goals related to the Triple Aim (acknowledging that realizing the benefit of digital tools is never “easy”).

In the demand-pull scenario, teams have generally already agreed upon the perceived value of a particular tool for solving a problem they face, and are ready to engage in service changes in order to put the technology to use. However, in the technology-push scenario, the value of the digital tool may be entirely unclear to the team of people who are expected to use it. This observation raises two additional concepts that are frequently used during the process of service design: “pains” and “gains”. In this sense, *pains* refer to “bad outcomes, risks and obstacles related to customer jobs”,^7^ which are negative issues that arise during the normal course of completing work-related tasks. *Gains* refer to “outcomes customers want to achieve or the concrete benefits they are seeking”.^7^ Often in demand-pull scenarios, teams already understand the pains they are trying to solve and the gains they are hoping to achieve. Conversely, in technology-push scenarios, teams have not uncovered any pains and gains with clarity.

Both of these scenarios stand to benefit from a systematic process in which a new digital tool is introduced to the team, and the potential implications of the tool for their everyday work are examined, potentially altered, and eventually agreed upon. We suggest that methods of value proposition design, incorporating the principles of service design outlined earlier in this paper, present a clear process for identifying, modifying, and eventually acting upon the value of a new digital tool in health services contexts; this process represents a fundamental component of the earliest stages of implementation. We now turn to describing how value proposition design can inform the implementation process.

## Value proposition design for technology-enhanced services

Considering the complexity of the effort to identify a clear value proposition of a new digital tool, and to integrate that tool into processes of service delivery,^20^ it can be challenging to identify a useful place to begin.^24^ One fruitful entry point is through the concept of “value proposition design” (VPD).^7^

VPD is a methodology for establishing the actual value of a new product or process for the variety of people with whom that product or process interacts.^7^ Developers of new digital tools generally have a clear opinion about the value of their product, but that value is likely not interpreted the same way by every patient, clinician, and payer.^25^ VPD encourages teams to be brutally honest in examining the *actual value* that a new technology might have for their service, understood through solutions to their pains and potential newly added gains, and to introduce modifications where feasible to make the technology more valuable for their needs. This is where the fourth principle of service design stated earlier becomes central: committing to a series of iterative learning cycles that introduce changes to the technology or the way it fits within an emerging picture of the newly established service.

The VPD approach raises a central point about the application of service design to the introduction of new technologies in health care: The successful deployment of a technology that advances the achievement of the Triple Aim relies on a collection of people seeing *value* in the new technology and as a result building meaningful changes into their everyday work routines. A randomized trial examining the effectiveness of a technology is thus never just examining the technology itself; it is examining how well the technology “enlists” health care providers, managers, patients, and others to see value in a new way of doing things.^26^ This insight, that a technology is only one component of an overarching program or intervention in health care, highlights the importance of clear value propositions to *all users* and the need to consider a broader range of issues than the technology itself.

Investigating the specific value propositions that a technology may have for health care providers (by assessing its impact on their “pains and gains”), and the value propositions that may be generated for users through the development of a newly configured service, raises the issue of the *goals* of implementation. Value proposition design encourages an approach that makes it as easy as possible for users to adopt a new technology, leading to an emphasis in health care settings on the importance of minimal changes to workflow for clinicians. However, this adoption goal must be balanced against the transformational orientation of many service design approaches, which often seek to enable more complete changes to the organization of services in order to promote better service experiences and outcomes for patients. It is this latter goal, to re-orient the routines of health service providers for more creative and effective services, that constitutes the more challenging objective of service design and implementation science. Acknowledging this point, we now turn to describe a simple heuristic that can provide insight into the reasons why such change may be so challenging, and a structured approach to achieve it.

## A simple heuristic for technology-related service design

Building on insights from VPD, we suggest that the intervention being evaluated upon the introduction of a new technology is never simply the technology itself. The intervention being evaluated is the *new service* being established by the interactions between a new tool (generally a technology), a team of health care providers and other stakeholders, and newly established routines of service delivery.^27–29^ Here we introduce our simple heuristic for representing this collection of considerations that make up the intervention: [Tool+Team+Routine].

The heuristic [Tool+Team+Routine] is grounded in a variety of theories and frameworks about the introduction of technologies into health care environments, and especially the Fit between the Individual, Task and Technology (FITT) Model,^30,31^ the ARCHIE framework on technology design and implementation,^32^ and normalization process theory.^33^ Although each of these approaches is conceptually sound, we have found their application in actual contexts of technology adoption projects to be contested and challenging. As a result, we developed the heuristic [Tool+Team+Routine] to incorporate these perspectives into an approach that is primarily focused on service design, arising from seminal works in this domain.^7,9,20^

The purpose of this heuristic is not to introduce new concepts into the field of technology adoption, but instead to integrate existing concepts in a way that presents a *concise and useful reminder* of the central considerations to making new technologies work in health service environments during the early stages of implementation. Each of the three components of [Tool+Team+Routine] is fundamental to the service innovations arising from the introduction of a new technology, and each deserves attention during the service design and implementation processes.

In the next section, we present an illustrative scenario of how the [Tool+Team+Routine] heuristic might be applied to the deployment of a digital care coordination platform in a particular health care delivery context, and provide a summary figure depicting the heuristic. The scenario emphasizes the variety of people whose needs and interests are usefully considered when engaging in service design activities, and highlights the fact that the intervention is not just the technology, but is instead constituted by each of these components working in concert.

One additional point is necessary prior to presenting our case scenario. The [Tool+Team+Routine] heuristic is primarily focused on the adoption of technologies for service change, and thus the level of analysis is located within an organization at the level of a team, ward, or other clinical group unit. This level of focus is essential for understanding adoption processes and the service changes that arise, but leaves out an essential component of the technology adoption process: *Procurement*. Addressing procurement is beyond the scope of this paper, but is addressed in forthcoming work by our research team.

## Illustrative scenario of [Tool+Team+Routine] heuristic

Here we present an example of a digital care coordination platform being introduced to enhance the case management of patients with complex health needs in the community. The platform enables health care providers to exchange information about specific patients, engage in inter-professional dialogue where appropriate, and thereby enhance the overall management of each patient. Whether the digital platform is introduced with a “demand-pull” or “technology-push” scenario, the heuristic [Tool+Team+Routine] helps to identify the key questions and issues requiring attention in the effort to promote an effective and coordinated service.

### Tool

The first question recommended by the [Tool+Team+Routine] approach relates to whether there is a clearly stated value proposition for all those who must interact with the digital platform. Do physicians, care coordinators, health care administrators, and community pharmacists see how the platform will enhance their ability to care for patients? Not only that, but does the platform provide an opportunity to reduce their workload, or does it simply add responsibilities that did not previously exist? These are crucial questions to answer at the very beginning of a service design project.

Additional issues related to the digital tool that are raised by a service design approach include the way it will interface with other technologies (or not), whether the vendor is willing to make modifications to better fit the tool into the specific context, and whether all types of users have already trialed the tool during development. These provide crucial insight into how robustly the tool has been developed, and how it will fit into the particular context of service delivery.

### Team

Questions related to the team of people who must interact with the technology emphasize the point that service design projects are less about the digital tool being deployed and more about that actual people expected to use it. Two key starting points relate to (a) whether the team has all agreed that there is a problem worth solving (i.e., whether this is a demand-pull versus technology-push project), and (b) what implications the digital tool has for relationships among team members. Does the care coordination platform mean family physicians will need to interact with community pharmacists? Have they ever interacted before? Will they need to develop new relationships? These are issues raised by the emphasis on the team of people involved in a service design project, and point toward the areas of project management that will require most attention if the service design project is the be successful.

### Routine

The focus on routines of care emphasizes the point that people work in routinized ways in everyday environments. Even though health care work is highly skilled and involves a variety of forms of expertise, health care workers nonetheless function through a series of work-related routines. This includes the work that physicians must do to record patient information and communicate with other health care providers, but it also includes the routines required by staff responsible for procuring and maintaining a given digital tool. How is the digital tool being procured, and what changes might be required to the work of people responsible for sourcing, justifying, and purchasing the tool? The “routine” component of [Tool+Team+Routine] emphasizes that routines extend beyond the act of care delivery to include those who support the sustainability of the service overall. Bringing all of the stakeholders together who are implicated by the adoption of a new digital tool, and engaging in discussion about how work routines will change, is a central component of successful service innovation in health care settings.

### Summary

**Fig. 1:**
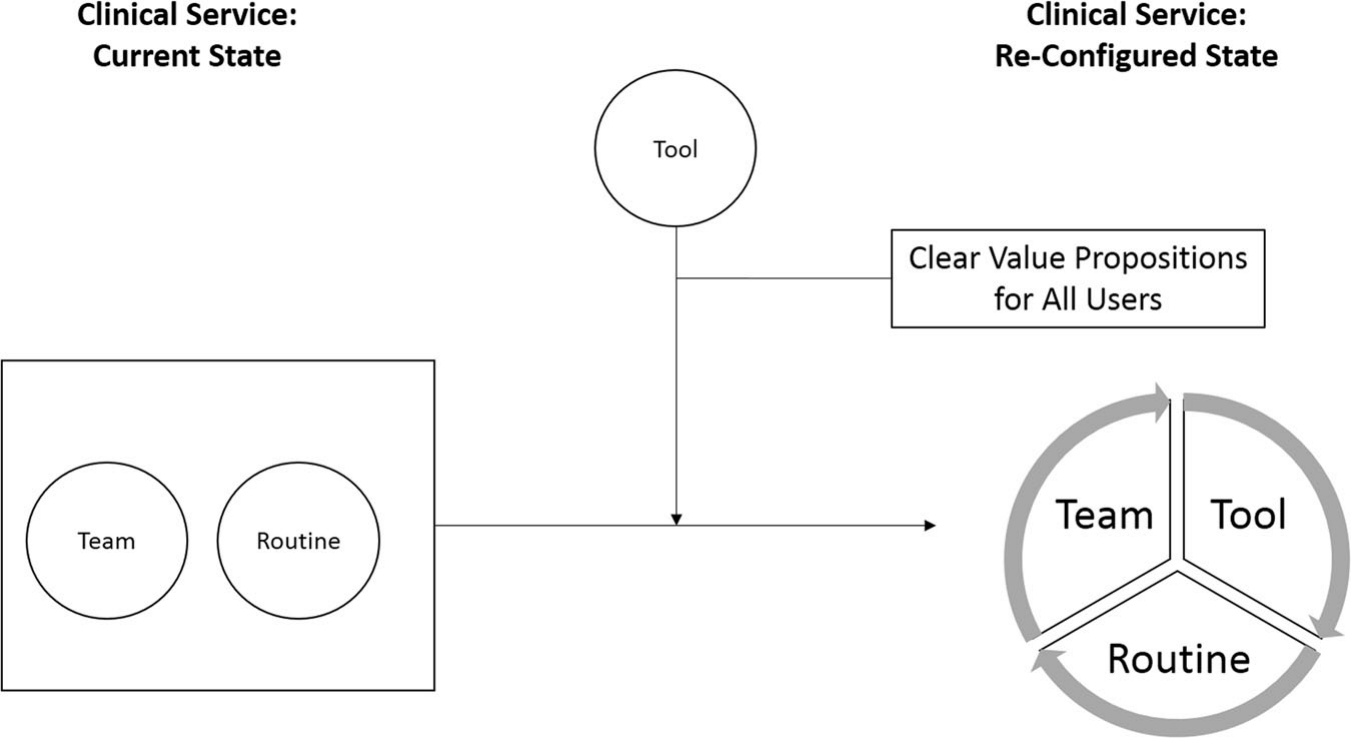
Team+Tool+Routine. When a technology (tool) includes a clearly stated, meaningful value proposition for all users who must interact with the technology or the information it generates, the team can use the technology to establish new routines involved in providing care, and ultimately a re-configured service

In this case example, the [Tool+Team+Routine] heuristic proved fundamental to identifying the key stakeholders who would be implicated in the technology implementation, determining the value propositions offered by the technology (which informed our selection of implementation outcome measures), and articulating which routines would require changing during the adoption process. The heuristic gave the implementation and research teams enhanced clarity about what challenges would arise, and structured our approach to dialogue with health care providers to determine whether they could engage with the technology in meaningful ways. By clearly articulating the new routines required, the team was successful in identifying providers to be involved in early-stage service design, developing a stronger understanding of effective implementation approaches as the project progressed (Fig. 1).

## Conclusion

The concepts introduced here provide a starting point for shifting thinking away from conventional approaches to implementing technologies and moving toward a more comprehensive approach to service design. Viewing technology adoption as an iterative process, involving complex interactions between a tool, a team, and newly established routines, stands to help teams envision new services arising from the adoption of technologies beyond the added work of new forms of data entry and communication. However, two challenges in particular remain.

The first challenge that will need to be addressed as service design approaches become increasingly used during implementation relates to the challenge of envisioning a future configuration of health services and encouraging the changes to daily routines that are necessary to realize that future. Value proposition design offers a strategy by which teams can identify the benefits of new technologies for clinical teams, but taking teams out of their comfort zones toward enacting more comprehensive service innovations requires creativity and bold leadership. Effective strategies that represent such creativity and leadership are in high demand, and this demand will continue to grow over time.

The second challenge relates to establishing the evidentiary base that is required to garner large-scale support for service design approaches during the implementation process. Much as implementation science has developed as a legitimate field of research incorporating a broad collection of methods and theories, the same will need to happen with service design (acknowledging the substantial base of literature that already exists on the topic). This is not just about generating evidence of the effectiveness of service design as an approach to implementation, but is about imagining new approaches and applications that will drive the discipline of service design in health care forward. For the time being, continued conceptual dialogue about the fit between service design and conventional approaches to implementation science will help to lay the groundwork for the important advances on this topic yet to come.
